# Menstrual blood-derived endometrial stem cell, a unique and promising alternative in the stem cell-based therapy for chemotherapy-induced premature ovarian insufficiency

**DOI:** 10.1186/s13287-023-03551-w

**Published:** 2023-11-13

**Authors:** Shenghui Zhang, Badrul Hisham Yahaya, Ying Pan, Yanli Liu, Juntang Lin

**Affiliations:** 1 Stem Cell and Biotherapy Technology Research Center, Henan Joint International Research Laboratory of Stem Cell Medicine, Xinxiang Medical University, East of JinSui Road, Xinxiang, Henan China; 2 Lung Stem Cell and Gene Therapy Group, Regenerative Medicine Cluster, Advanced Medical and Dental Institute (IPPT), Universiti Sains Malaysia, Penang, Malaysia; 3 The Third Affiliated Hospital of Xinxiang Medical University, Xinxiang, ‬ China

**Keywords:** Chemotherapy, Premature ovarian insufficiency, Mesenchymal stem cells, Menstrual blood-derived endometrial stem cells, Paracrine effect, Fertility

## Abstract

Chemotherapy can cause ovarian dysfunction and infertility since the ovary is extremely sensitive to chemotherapeutic drugs. Apart from the indispensable role of the ovary in the overall hormonal milieu, ovarian dysfunction also affects many other organ systems and functions including sexuality, bones, the cardiovascular system, and neurocognitive function. Although conventional hormone replacement therapy can partly relieve the adverse symptoms of premature ovarian insufficiency (POI), the treatment cannot fundamentally prevent deterioration of POI. Therefore, effective treatments to improve chemotherapy-induced POI are urgently needed, especially for patients desiring fertility preservation. Recently, mesenchymal stem cell (MSC)-based therapies have resulted in promising improvements in chemotherapy-induced ovary dysfunction by enhancing the anti-apoptotic capacity of ovarian cells, preventing ovarian follicular atresia, promoting angiogenesis and improving injured ovarian structure and the pregnancy rate. These improvements are mainly attributed to MSC-derived biological factors, functional RNAs, and even mitochondria, which are directly secreted or indirectly translocated with extracellular vesicles (microvesicles and exosomes) to repair ovarian dysfunction. Additionally, as a novel source of MSCs, menstrual blood-derived endometrial stem cells (MenSCs) have exhibited promising therapeutic effects in various diseases due to their comprehensive advantages, such as periodic and non-invasive sample collection, abundant sources, regular donation and autologous transplantation. Therefore, this review summarizes the efficacy of MSCs transplantation in improving chemotherapy-induced POI and analyzes the underlying mechanism, and further discusses the benefit and existing challenges in promoting the clinical application of MenSCs in chemotherapy-induced POI.

## Introduction

Generally, the age at physiological menopause is approximately 50 years, and ovarian function decline is a progressive process. When women experience menstrual abnormalities, FSH levels rise and estrogen volatility decreases before 40 years of age is defined as premature ovarian insufficiency (POI) [1]. The terminal stage is premature ovarian failure (POF), which is mainly characterized by amenorrhea, follicle-stimulating hormone (FSH) levels > 40 IU/L, reduced estrogen levels and varying degrees of perimenopausal symptoms and infertility [2]. However, with the current trend toward a younger onset of cancer and the widespread use of radiotherapy and chemotherapy, although the success rate of cancer treatment in children, adolescents and women of childbearing age has increased, iatrogenic ovarian function damage caused by chemotherapy drugs is a growing concern, especially in patients with fertility needs [3]. The ovaries are very sensitive to chemotherapeutic drugs, especially alkylating agents, leading to severe gonadal dysfunction. Studies have shown that cyclophosphamide (CTX) causes the greatest damage to oocytes and granulosa cells (GCs) in a dose-dependent manner, while the combined use of abdominal ionizing radiation and alkylating agents induce POF, resulting in infertility in approximately 100% of patients [4–6]. Furthermore, pediatric cancer research shows that among survivors who received alkylating agents combined with abdominal ionizing radiation, the cumulative incidence of premature menopause was close to 20% [7, 8]. Therefore, although tumor treatment can extend the survival rate of patients, the sequelae of ovarian dysfunction will also lead to early menopause and loss of fertility, which is closely related to the occurrence of hot flashes, osteoporosis and cardiovascular disease [9–11]. Currently, the main treatment for POF is hormone replacement therapy (HRT) [12]. However, HRT can only relieve perimenopausal symptoms and cannot fundamentally prevent POF [13]. In addition, long-term administration of exogenous hormones can significantly increase the occurrence of thrombotic diseases and tumors [14, 15]. Furthermore, for infertile patients, reproductive can be fulfilled only by methods such as oocyte cryopreservation, embryo transplantation, and ovarian tissue cryopreservation, and delaying cancer treatment to maintain fertility and acquire oocytes is considered unacceptable [16, 17].

Fortunately, stem cell-based therapies result in promising improvements in diseases without effective treatments. A variety of stem cells, including induced pluripotent stem cells (iPSCs), mesenchymal stem cells (MSCs) and endothelial progenitor cells (EPCs), especially MSCs, have been used to treat animal models with chemotherapy-induced POI, and promising improvements have been clearly observed after stem cell transplantation [18]. In addition, after decades of research, menstrual blood-derived endometrial stem cells (MenSCs) have exhibited promising therapeutic effects in diseases lacking effective treatment due to their comprehensive advantages, such as periodic and non-invasive sample collection, abundant sources, stable donation, and autologous transplantation [19, 20]. This review is to summarize the efficacy of MSCs transplantation in improving chemotherapy-induced POI, analyze the underlying mechanism, and further discuss the benefit and existing challenges in promoting the clinical application of MenSCs in chemotherapy-induced POI.

## Therapeutic effects of MSCs transplantation on chemotherapy-induced POI

Many preclinical and clinical studies have confirmed the therapeutic potential of MSCs transplantation for POI in the past decades. As shown in Table 1, MSCs transplantation enhances the anti-apoptotic capacity of ovarian-associated cells and promotes regeneration of these cells, especially GCs and primordial germ cells (PGCs). Moreover, angiogenesis and stromal injury restoration in impaired ovaries are significantly improved after MSCs transplantation, which is partly attributable to regulating reactive oxygen species (ROS) production. Consequently, MSCs transplantation likely restores impaired ovarian function at both the cellular and tissue levels.

**Table 1:** Preclinical animal trials using MSCs to treat POI

Animal model	Stem cell type (or derivatives)	Dosage	Delivery route	Results	References
CTX-induced Wistar–Imamichi rats	Adipose-derived MSCs (ADSCs)	2 × 10^6^ cells	Intraovarian injection	ADSCs transplantation could induce angiogenesis and restore the number of ovarian follicles and corpus lutea in ovaries	[21]
CTX-induced C57/BL6 mice	ADSCs	1 × 10^6^ cells, (Intravenous); 1 × 10^5^ cells, (Intraovarian)	Intravenous and intraovarian injection	ADSCs transplantation could significantly upregulate the population of follicles at different stages and ovulation	[22]
CTX-induced white albino rats	Amniotic membrane-derived MSCs (AMMSCs) and ADSCs	5 × 10^6^ cells	Intravenous injection	Both AMMSCs and ADSCs transplantation exert a significant therapeutic efficacy in chemotherapy-induced ovarian insult in rats; and AMMSCs transplantation exert higher therapeutic efficacy when compared to ADSCs	[23]
CTX-induced Wistar rats	Rat bone marrow-derived mesenchymal stem cells (BMSCs) overexpressing miR-21	1 × 10^6^ cells	Intraovarian injection	After BMSCs transplantation, the ovarian weight and follicle counts increased; estradiol levels increased while FSH levels decreased, with less severe apoptosis of GCs	[24]
CTX-induced Sprague–Dawley (SD) rats	LIPUS-pretreated human AMMSCs	4 × 10^6^ cells	Intravenous injection	LIPUS-pretreated AMMSCs transplantation is more advantageous for reducing inflammation, improving the local microenvironment, and inhibiting GC apoptosis induced by chemotherapy	[25]
Cisplatin-induced C57BL/6 mice	MenSCs	2 × 10^6^ MenSCs on days 1 and 3 of the experiment	Intravenous injection	MenSCs transplantation could improve the ovarian microenvironment by reducing apoptosis in GCs and the fibrosis of ovarian interstitium, which contributes to increase the follicular numbers and return sex hormone levels to normal values	[26]
CTX-induced Wistar rats	Rat BMSCs	1 × l0^6^ cells	Intraovarian injection	heat shock-pretreated BMSCs transplantation could cause an increase in ovary weight and the number of follicles at different stages of estradiol levels; and a decrease in FSH levels and apoptosis of GCs	[27]
CTX-induced C57/BL6 mice	Human chorionic plate-derived mesenchymal stem cells (hCPMSCs)	2 × l0^6^ cells/kg	Intravenous injection	hCPMSCs transplantation restored the serum hormone level and ovarian function of CTX-induced POF mice	[28]
CTX-induced C57BL/6 mice	Menstrual Blood-Derived Stromal Cells	1 × 10^6^ cells	Intravenous injection	Menstrual Blood-Derived Stromal Cells restore ovarian function by regulating normal follicle development and estrous cycle via regulating the ECM-Dependent FAK/AKT Signaling	[29]
Cisplatin-induced C57BL/6 mice	BMSC-derived exosomes	125 μg of exosomal proteins on the 1st, 5th, and 10th day after modeling	Intravenous injection	BMSC-derived exosomes improved the follicular morphology of POF mice and inhibited the expression of apoptosis-related protein in vivo; furthermore, BMSC-derived exosomes repressed cisplatin-induced GCs apoptosis and increased cells viability in vitro	[30]
Busulfan and CTX-induced ICR mice	UCMSC-derived microvesicles (UCMSC-MVs)	150 μg	Intravenous injection	UCMSC-MVs treatment could increase the body weight and number of ovarian follicles (primordial, developing, and preovulatory follicles), induce ovarian angiogenesis and recover the disturbed estrous cycle of POI mice	[31]
10% hydrogen peroxide induced BALB/c mice	Human AMMSCs	1 × 10^6^ cells	Intraperitoneal injection	The estrus cycle was recovered after hAMSCs transplantation at 7 and 14 days. Estrogen levels increased, while FSH levels decreased. The ovarian index, fertility rate, and population of follicles at different stages were significantly increased. The newborn mice had no obvious deformity and showed normal growth and development. The normal offspring mice were also fertile	[32]
CTX-induced ICR mice	Fetal liver-derived MSCs (fMSCs)	1 × 10^6^ cells	Intravenous injection	fMSCs transplantation could prevent CTX-induced follicle loss and recover sex hormone levels; significantly decrease oxidative damage, increase oxidative protection; enhance anti-apoptotic effects and inhibit apoptotic genes in vivo and in vitro	[33]
CTX-induced SD rats	BMSCs and BMSC-derived exosomes	1 × 10^6^ cells every other day for 2 weeks; 150 μg of exosomal proteins	Intraperitoneal injection	Both BMSCs and BMSC-derived exosomes transplantation could significantly recover the estrus cycle, increase the number of basal and sinus follicles; and increase estradiol and anti-Mullerian hormone (AMH) levels, but reduce FSH and luteinizing hormone levels in serum	[34]
CTX-induced SD rats	Amniotic fluid mesenchymal stem cells (AFSCs); AFMSCs-derived extracellular vesicles (AFMSCs-EVs)	5 × 10^5^ cells, 100 μg	Intraovarian injection	AFMSCs and AFMSC-EVs treatment equally restored total follicular counts, AMH levels, regular estrous cycles and fruitful conception, while it both diminished caspase 3 and PTEN levels	[35]
Surgically removing one of the ovaries in SD rats	Placenta-derived mesenchymal stem cells (PDMSCs)	5 × 10^5^ cells	Intravenous injection	PDMSCs transplantation could significantly increase the levels of AMH, FSH, and estradiol; and more mature follicles, less atresia and restoration of expanded blood vessels in the ovaries of PDMSCs treated rat	[36]
Surgically removing one of the ovaries in SD rats	PDMSCs	5 × 10^5^ cells	Intravenous injection	The levels of apoptotic factors were decreased and ovary function was improved following PDMSCs transplantation	[37]
Busulfan and CTX-induced C57BL/6 mice	UCMSCs with overexpressing HO-1	1 × 10^6^ cells	Intraperitoneal injection	HO-1 overexpressed UCMSCs transplantation could recover the ovarian function, increase GCs’ viability and decrease their apoptosis	[38]
Cisplatin-induced ICR mice	Human embryonic stem cell-derived MSCs (hESC-MSCs)	5 × 10^6^ cells	Intravenous injection	The primary and primordial follicle counts in the ovaries of hESC-MSC treated group were significantly improved, and the count of zona pellucida remnants was significantly reduced	[39]
CTX-induced C57BL/6 mice	UCMSC-derived exosomes (UCMSC-Exos)	10^12^ particles/ml	Intraovarian injection	UCMSC-Exos inhibited apoptosis of CTX-injured human GCs, alleviated oxidative stress and rescued ovarian phenotype and function	[40]
Busulfan and CTX-induced C57BL/6 mice	hESC-MSCs and BMSCs	1 × 10^6^ cells respectively	Intravenous injection	hESC-MSCs were similar to BMSCs in that they could restore the structure of the injured ovarian tissue. Meanwhile, hESC-MSCs promoted of follicular development, fertility via a paracrine effect	[41]
CTX-induced Wistar rats	MenSCs	2 × 10^5^ cells/10 μl	Intraovarian injection	CD 146 + MenSCs transplantation increased the number of developing follicles, decreased the number of atresia follicles, and improved ovarian fibrosis	[42]
4-vinylcyclohexene diepoxide- induced SD rats	MenSCs and MenSC- derived exosomes	5 × 10^5^ MenSCs; 25 μg MenSCs- exosomes	Intraovarian injection	MenSCs- derived exosomes promotes follicular development, activates dormant follicles, and improves POI rats’ fertility	[43]
CTX-induced C57BL/6 mice	UCMSCs	5 × 10^5^ cells	Intraovarian injection	UCMSCs promoted granulosa cell proliferation and ovarian vascularization	[44]
Busulfan and CTX-induced C57BL/6 mice	UCMSCs	2 × 10^6^ cells	Intravenous injection	Multiple UCMSCs transplantations have a better effect on the recovery of ovarian function than single hUC-MSC transplantation in POF	[45]
Busulfan and CTX-induced C57BL/6 mice	UCMSCs	1 × 10^6^ cells	Intravenous injection	UCMSCs transplantation improve ovarian function through anti-apoptotic and anti-inflammatory effects via a paracrine mechanism	[46]
CTX-induced SD rats	human umbilical cord blood platelet-rich plasma (ucPRP) and UCMSCs	35 μL ucPRP with 2 × 10^6^ cells	Intraovarian injection	The combined application of HucMSCs and ucPRP increased the levels of serum E2, AMH, and FSH via promoted ovarian angiogenesis and proliferation and reduce the apoptosis of ovarian granulosa cells	[47]
CTX-induced C57BL/6 mice	hESC-MSCs	1 × 10^6^ cells	Intravenous injection	hESC-MSCs reduced apoptosis in the follicles and increased the expression of AMH protein	[48]

### Effects of MSCs transplantation on chemotherapy-induced ovarian cell injury

#### Granulosa cells (GCs)

Follicles, which are the functional units of the ovary, mainly include oocytes, the surrounding GCs, and theca cells [49]. GCs play a key role in ovarian function during the maturation and release of oocytes by regulating hormone production [50]. At the embryonic stage, the number of oocytes in embryonic ovaries peaks. After birth, the oocytes are still in the early stage of the first meiosis and are surrounded only by a layer of flat GCs. In adolescence, the primordial follicles are recruited. They are closely related to bone growth and mineralization, sudden growth, secondary sexual characteristics (mammary development, pubic hair development), cardiovascular and nerve changes [51, 52]. Therefore, chemotherapeutic drugs have a tremendous impact on ovarian function damage. The chemotherapeutic drugs with the highest gonadal toxicity are nitrogen mustard-derived alkylating agents (such as CTX), followed by platinum analogs, taxanes, plant alkaloids and anthracyclines [53–58]. These chemotherapy drugs mainly activate the apoptotic pathway by releasing cytochrome C from mitochondria to induce DNA damage, thereby killing cells with high proliferative activity. Therefore, while killing tumor cells, chemotherapy drugs may also accidentally impair some cells with physiologically high proliferative activity, such as ovarian GCs, which are highly dependent on interaction with oocytes during oocyte maturation [59–63]. Ovarian GCs are rich in mitochondria, and play a nutritional and supportive role in developing oocytes; excessive apoptosis of GCs can inhibit follicular development and induce follicular atresia, thereby affecting fertility in women [64].

Therefore, enhancing the antiapoptotic capacity of GCs or promoting GCs regeneration can restore the function of GCs, effectively improving chemotherapy-induced ovarian damage. A previous study found that intraovarian injection of red fluorescent protein (RFP)-labeled human amniotic fluid cells (AFCs) resulted in RFP-positive CD44^+^CD105^+^ HuAFCs in POF mice at the injection site in the ovary within 3 weeks, indicating that MSCs can remain in the damaged ovary for a long period and thus providing a theoretical basis for the use of MSCs in the treatment of POF [65]. In addition, as intercellular messengers, MSC-derived exosomes can participate in ovarian function improvement by preventing GC apoptosis. In a cisplatin-induced POF model, umbilical cord mesenchymal stem cell (UcMSC)-derived exosomes (UcMSC-Exos) have been reported to significantly upregulate the expression of the antiapoptotic protein Bcl-2 in GCs and downregulate the expression of the proapoptotic protein caspase-3, thereby protecting GCs from apoptosis [66]. Moreover, exosomes derived from bone marrow stromal cells (BMSCs) also show similar beneficial effects, and overexpression of miR-664-5p in BMSC-derived exosomes has been reported to target the p53 signaling pathway to ameliorate cisplatin-induced damage to GCs. In contrast, of miR-664-5p in BMSCs can reduce the protective effect on GCs conferred by BMSCs-derived exosomes [30]. Subsequently, miR-144-5p in BMSC-derived exosomes was reported to inhibit CTX-induced GC apoptosis by targeting phosphatase and tensin homolog deleted on chromosome 10 (PTEN) to activate the phosphatidylinositol-3-kinase (PI3K)/protein kinase B (Akt) signaling pathway [34]. Furthermore, researchers have found that heat shock-treated BMSCs were more tolerant to the microenvironment of chemotherapy and could significantly enhance the therapeutic effect of BMSCs on chemotherapy-induced POF [27]. In addition, hemeoxygenase-1 (HO-1)-overexpressing UcMSCs transplantation could inhibit GCs apoptosis and improve ovarian function by regulating autophagy through activation of the JNK/Bcl-2 signaling pathway, and increased levels of HO-1 may also improve ovarian metabolic disorders by upregulating the circulation of CD8^+^CD28^−^ T cells to suppress inflammation [67].

#### Primordial germ cells (PGCs)

Chemotherapeutic drugs-induced DNA damage, such as double-strand breaks (DSBs), inter- and intra-strand crosslinks, intercalation, and monoalkylation, can significantly induce apoptosis of follicular cells and cause follicular atresia, which leads to follicular depletion [68, 69]. Resting germ cells are more sensitive to cell cycle non-specific drugs, such as alkylating agents and topoisomerase inhibitors, and the DNA damage caused by chemotherapeutic drugs can trigger apoptosis of PGCs mediated by TAp63 [70, 71]. In a CTX-induced POF mouse model, AFSCs transplantation significantly increased the number of primordial follicles, and the number of atretic follicles significantly decreased, suggesting that AFSCs can differentiate into primitive oocytes and promote follicle formation in injured ovaries [72]. As further verification of the role of MSCs in the formation of primordial follicles, subsequent studies showed that after transplantation of iron oxide-labeled BMSCs, Prussian blue stain-positive cells could be observed in newly formed follicles, indicating that MSCs transplantation may be directly involved in the formation or regeneration of primordial ovarian follicles. After BMSCs transplantation, female mice can have a normal pregnancy and give birth to healthy mice, indicating that new follicles generated by BMSCs transplantation have normal functions [73]. Moreover, studies have shown that amniotic mesenchymal stem cell (AMSCs) transplantation can not only activate endogenous ovarian germ stem cells to promote their differentiation into primordial follicles, but also improve the ovarian microenvironment to restore its function [32]. In addition, MSCs may also restore ovarian function by improving germ cell development and reducing primordial germ stem cell depletion. However, the underlying mechanism remains unclear.

### Improvement of chemotherapy-induced vascular injury by MSCs transplantation

Vascularization is a prominent feature of tissue formation and is responsible for the supply and assurance of oxygen, nutrients and signal transduction in tissues and cells [74]. Ovarian stromal vessels start to proliferate in the secondary follicle stage and form a network of capillaries around the follicles, providing nutrition and ensuring intercellular communication for normal ovarian function, especially in the superficial ovarian cortex, because resident primordial follicles and early growing follicles do not have an independent vascular network; therefore, the superficial ovary cortex is highly dependent on interactions with stromal vessels [75]. However, ovarian blood flow is dramatically reduced after doxorubicin injection, and severe vascular wall disintegration occurs. Ultrasound biomicroscopy with microbubbles has shown that doxorubicin has an acute toxic effect on ovarian blood vessels, and ovarian blood flow decreased by 33% after 3 min of injection. In addition, doxorubicin was further demonstrated to cause acute damage to ovarian microvessels, resulting in an irregular vascular wall and a significant decrease in microvessels [76].

A study has shown that PDMSCs transplantation could significantly upregulate the expression of vascular endothelial growth factor (VEGF) and VEGF receptor 2 in the ovaries of ovariectomised rat model through PI3K/AKT/mTOR and GSK3β/β-catenin pathway activation [36]. The above results indicated that MSCs transplantation likely promoted ovarian vascular regeneration through significant upregulation of VEGF expression, leading to ovarian dysfunction repair. In addition, further studies showed that UcMSC-microvesicles (MVs) transplantation could upregulate the expression of angiogenic factors such as VEGF through activation of PI3K-Akt signaling pathway, thereby inhibiting endothelial cell apoptosis and promoting angiogenesis, which results in significantly increased vascular density and CD34^+^ cells in the ovaries, ultimately improving ovarian function [31].

### Improvement of chemotherapy-induced ovarian stroma injury by MSCs transplantation

The superficial cortical stroma of the ovary constitutes the living microenvironment of most primordial follicles. Activation of a primordial follicle and its entry into the growth phase as a primary follicle, and the transition from primary to secondary follicles, occur in the superficial cortical stroma of the ovary. The cortical stroma is composed of fusiform and fibroblast-like cells, which are densely packed in the ovaries. Cortical stromal cells are the supportive cells of resting and early growing follicle and the source of theca cells [77, 78]. The close geographical relationship between resting and early growing follicles and the superficial cortical stroma provides the material interaction basis between follicles and cortical stromal cells. Stromal cell injury is considered an intermediate step leading to POF. In addition to the direct exposure of follicles and oocytes to chemotherapeutic drugs, stromal cell injury may directly disrupt endocrine homeostasis in patients [79]. In a clinical study on successful treatment of pediatric leukemia, further ultrastructure examination of ovarian tissue showed that after chemotherapy, children had obvious ovarian cortex fibrosis accompanied by conditions such as transparency, calcification and an absence of follicles, and the normal structure of the matrix was disrupted by a large number of collagen bundles [80].

In a busulfan-induced POF mouse model, GFP-labeled MenSCs were injected through the tail vein, and the results showed that most of the MenSCs migrated to the ovarian stromal area [81]. Similarly, PKH26-labeled human adipose-derived mesenchymal stem cells (ADSCs) were transplanted into injured ovaries, and the results indicated that the ADSCs mainly localized in ovarian stromal tissues seven days after transplantation, suggesting that ADSCs most likely do not directly differentiate into follicular cells or GCs but improve impaired ovarian function by secreting various biological factors, such as tissue growth factor (TGF)-β, hepatocyte growth factor (HGF), insulin-like growth factor (IGF)-1, VEGF, epidermal growth factor (EGF) and basic fibroblast growth factor (bFGF), and these factors may promote the repair and regeneration of stromal cells through anti-apoptosis and inhibition of scar formation [21, 25]. Simultaneously, after AMSCs transplantation into autografted ovaries, the total volume of the ovary, cortex and medulla in animal model were significantly increased [82]. Furthermore, the transplanted MenSCs could directly migrate to the ovarian stroma to exert repair function and reduce ovarian stromal fibrosis, demonstrating that MSCs can repair ovarian dysfunction by improving the local microenvironment [26].

### Improvement of chemotherapy-induced ovarian oxidative damage by MSCs transplantation

Oxidative stress is caused by the production of excessive intracellular ROS and reactive nitrogen species, which causes an imbalance between the oxidation system and antioxidant system [83]. Oxidative stress is closely related to tissue injury and aging and is also an important cause of chemotherapy-induced ovarian injury [84]. Studies have shown that MSCs can activate relevant pathways, such as the forkhead box O (FOXO), NAD(P)H quinone dehydrogenase 1 (NOQ1)/mitogen-activated protein kinase (MAPK), PI3K/Akt and nuclear factor E2–related factor 2 (Nrf2)-antioxidant response element (ARE) pathways, by secreting various biological factors, including HGF, IL-6, IL-8, VEGF, brain-derived neurotrophic factor (BDNF), leukemia inhibitory factor (LIF),and exosomes, and then improving ovarian function by increasing the production of antioxidant enzymes and inhibiting ROS production [85–92]. human PDMSCs transplantation can also exert an antioxidant effect and repair ovarian injury by downregulating the expression of oxidative stress markers (HO-1, HO-2) and upregulating the expression of antioxidant factors (superoxide dismutase and catalase) [37]. Furthermore, based on the sequencing results of UcMSCs exosomal miRNAs, Ding et al. [40] found that UcMSCs can inhibit SIRT7 expression by translocating miR-17-5p via exosomes to reduce ROS accumulation and restore ovarian function in a POI mouse model. In addition, a large number of studies have demonstrated that melatonin can bind to the melatonin membrane receptor (MT) to exert an antioxidant function and mediate cytoprotection [93]. In explorations of whether the antioxidant effect of MSCs is related to the MT, Huang et al. [33] found that fetal liver-derived-MSCs can upregulate the expression of MT1, c-Jun N-terminal protein kinase 1 (JNK1), proliferating cell nuclear antigen (PCNA), and 5′ AMP-activated protein kinase (AMPK) in luzindole-treated human GCs, and enhance the antioxidant effect of human GCs. However, in the same coculture condition with fetal liver-derived-MSCs, the expression of these proteins in MT1-knockdown human GCs treated with luzindole was downregulated, which inhibited the antioxidant effect indicating that the antioxidant effect of MSCs is closely related to MT1. Therefore, MSCs transplantation may target MT1 to repair the ovarian injury by restoring the balance of the oxidation-antioxidation system through activation of downstream antioxidant-related signaling pathways and inhibition of ROS production.

## MSCs improve chemotherapy-induced POI through paracrine effects

**Fig. 1:**
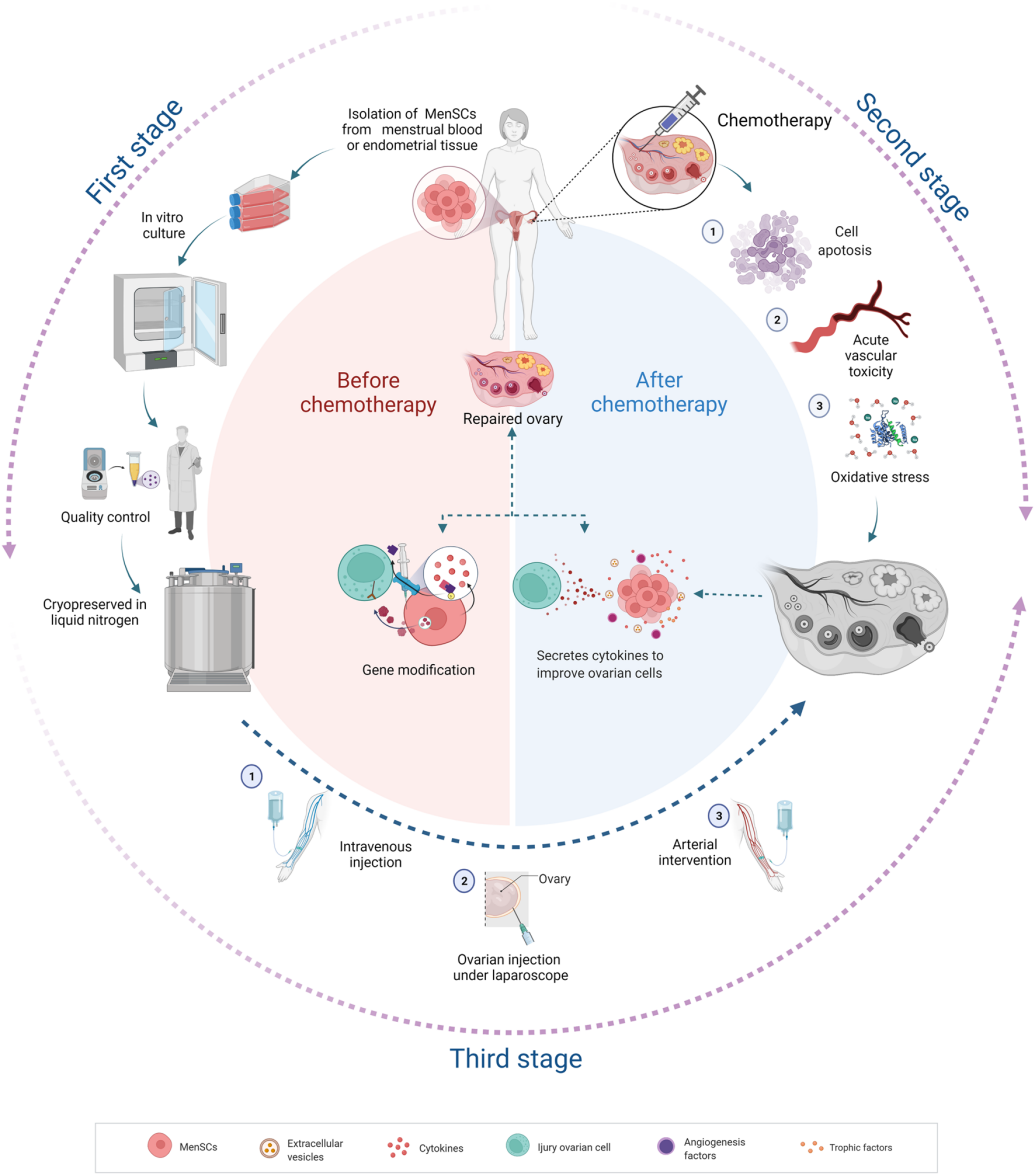
Autologous transplantation of MenSCs for improving chemotherapy-induced POI. Firstly, isolation of MenSCs from patients menstrual blood or endometrial tissue before receiving chemotherapy. After culture and amplification in vitro, MenSCs was cryopreserved in liquid nitrogen for further application. Subsequently, once the patient received chemotherapy, the chemotherapeutic drugs can cause ovarian toxicity mainly through impairing stromal tissue, oocytes, granulosa cells, and blood vessels, as well as aggravating oxidative stress which plays an important role in ovarian injury. Thirdly, after chemotherapy ends, the pre-cryopreserved autologous MenSCs can be transfused into the patient through intravenous injection, arterial intervention, and ovarian injection under laparoscopy. Finally, the transplanted MenSCs repair the ovarian dysfunction through enhancing the anti-apoptotic capacity of ovarian cells, preventing ovarian follicular atresia, promoting angiogenesis and improving injured ovarian structure. And these improvements are mainly attributed to MenSC-derived biological factors, functional RNAs, and even mitochondria, which are directly secreted or indirectly translocated with extracellular vesicles (microvesicles and exosomes) to repair ovarian dysfunction. The schematic diagram is created with BioRender.com

Previous studies have confirmed that pluripotent stem cells (embryonic stem cells (ESCs) and iPSCs) can be successfully differentiated in vivo and in vitro to produce functional GCs and promote the recovery of ovarian dysfunction [94–96]. MSCs also can differentiate into germ-like cells (such as GC-like cells and oocyte-like cells) under in vitro induction [97]. However, subsequent studies have shown that MSCs do not differentiate directly into germ-like cells in vivo but improve ovarian function through paracrine effects. Numerous studies have demonstrated that MSC-induced paracrine effects play a vital role in the improvement of diseases; therefore, improving ovarian function by MSCs also mainly depends on their superior paracrine effects [98]. Briefly, MSCs can target cells by secreting relevant biological factors, functional RNAs, and even mitochondria by direct secretion or through extracellular vesicles (microvesicles and exosomes) to improve chemotherapy-induced ovarian cell injury and restore ovarian function (Fig. 1).

### Biological factors

The biological factors secreted by MSCs include numerous soluble peptides and proteins, which can accelerate cell self-renewal, stimulate angiogenesis, inhibit cell apoptosis and alleviate inflammation. Current studies have shown that MSCs secrete a variety of growth factors, such as VEGF. IGF-1, HGF, FGF2, IL-6, IL-8, granulocyte colony-stimulating factor (G-CSF), stem cell factor (SCF), IL-11, IL-15, IL-10, platelet-derived growth factor (PDGF)-BB, and bFGF [99–102]. VEGF and IGF-1 can prevent cumulus cell injury and reduce the apoptosis of rat cumulus cells after cryopreservation [103, 104]. HGF mainly plays an antiapoptotic role in cumulus cells and theca cells [105]. FGF2 is considered necessary for angiogenesis, endometrial cell proliferation, and remodeling and plays an important role in repairing and regenerating damaged tissues [106].

### Extracellular vesicles

Extracellular vesicles can directly communicate with target tissue receptors or fuse with the plasma membrane to mediate information exchange between cells, participate in cell proliferation, migration and transformation, regulate the body's immune function, and affect angiogenesis and multiple biological processes [107]. Co-culture of UcMSCs-EVs with cisplatin-treated GCs has been reported to significantly downregulate caspase-3 mRNA expression and upregulate the Bcl-2/Bax ratio, thereby enhancing the ability of GCs to resist cisplatin-induced apoptosis [66]. Subsequently, after transplantation, UcMSCs-microvesicles (MVs) significantly upregulates AKT, p-AKT expression levels and angiogenesis-related cytokines in the ovary. Furthermore, UcMSCs-MVs are speculated to enhance the sensitivity of ovarian cells to IGF-1 via transmission of IGF1R, thereby inducing angiogenesis and ultimately promoting the recovery of ovarian function [31]. In addition, miR-17-5p is highly expressed in UcMSC-Exos, and UcMSCs can inhibit ROS accumulation by transmitting miR-17-5p in exosomes to inhibit SIRT7 expression, thus repairing ovarian dysfunction [40]. Consistently, several studies have shown that AFSCs-Exos inhibit GCs apoptosis by transmitting miR-146a and miR-10a, and evidence indicates that miR-644-5p inhibits cisplatin-induced apoptosis of ovarian GCs via targeted regulation of the p53 signaling pathway [30, 108].

### Gene engineering

Genetic modification can significantly enhance the paracrine effect of MSCs and promote their ability to repair ovarian dysfunction. Overexpression of miR-21 (a microRNA that regulates apoptosis) in BMSCs can inhibit the apoptosis of GCs by targeting programmed cell death protein 4 (PDCD4) and PTEN [24]. Similarly, overexpression of miR-144-5p in BMSCs resulted in significant improvement in a CTX-induced POF rat model, and this improvement was also associated with inhibition of GC apoptosis by targeting PTEN [34]. Additionally, transplantation of UcMSCs overexpressing HO-1 into POF mice promotes ovarian function recovery as discussed above [67].

## Benefit of cancer patient receive MSCs transplantation

MSCs transplantation have been extensively confirmed in promoting the repair and regeneration of tissues through performing anti-inflammatory action, secreting various nutritional cytokines and other distinguished mediators (extracellular vesicles, miRNA), which are beneficial to relieve the side effects of cancer patients received chemotherapy, and lay the foundation for taking MSCs-based therapy as an ideal auxiliary treatment to cancer patients [109–111]. Simultaneously, the safety of MSCs transplantation have also been testified and confirmed in various animal models and patients in clinic trials, mainly including tumorigenesis and pro-tumorigenic potential, and almost no report indicates that MSCs transplantation eventually causes tumor and promotes tumor proliferation and metastasis [112–115]. Unfortunately, no relatively clear interaction between MSCs and tumor cells are reported until now, especially whether exogenous MSCs transplantation potentially induce the cancer proliferation and recurrence, which play a crucial role in determining whether MSCs-based therapy could be used to improve the toxic and side effects caused by chemotherapy in cancer patients. Recently, although continuous studies attempt to reveal the role of exogenous MSCs in the occurrence, development and metastasis of tumors, the inconsistent conclusions were observed, and both MSCs-derived promotion and inhibition of tumor growth were reported [116].

### Controversy in MSCs transplantation for cancer patient

As mentioned above, MSCs secrete a variety of angiogenesis promoting factors, such as VEGF, PDGF, IL-6, IL-8, angiopoietin-1, HGF and BDNF, to improve the regeneration of blood vessels, which is reasonably postulated to promote the tumor growth [117, 118]. Beckermann et al. [119] have shown that BMSCs transplantation can promote the formation of tumor blood vessels through integrating into tumor blood vessels in nude mice model of pancreatic cancer xenotransplantation. This view was supported by Huang et al. [120] who pointed out that co-injection of human colorectal cancer cells and MSCs into nude mice could promote angiogenesis and tumor growth, resulting from MSCs-derived IL-6 induced the enrichment of proangiogenic factors secreted by cancer cells. Simultaneously, MSCs can also promote the metastasis of tumor cells by secreting cytokines and growth factors (HGF, PDGF, EGF, SDF-1 and TGF-β) [121–123]. An in vitro experiment shows that BMSCs can participate in the migration of neuroblastoma cells through stromal cell-derived factor-1 (SDF-1) /CXCR4 and CXCR7 signaling pathway [124]. Furthermore, a similar study confirms that BMSCs mixed with neuroblastoma cells significantly accelerated tumor growth and enhanced metastasis of neuroblastoma in vivo [125].

Oppositely, much more studies have demonstrated the role of MSCs in inhibiting the tumor growth and development, and it has been well documented that MSCs could inhibit the proliferation of tumor cells and upregulate the expression of apoptotic cytokines in tumor cells [126]. After 24 h of culture in 50% hUC-MSC-derived conditioned medium, the inhibition rate of human cholangiocarcinoma cells (HCCC-9810) increased from 6.21 to 49.86%, and the apoptosis rate increased from 9.3 to 48.1% [127]. Further research indicated that MSCs from the rib perichondrium significantly inhibit the growth, migration and invasion of the rat breast cancer cells (SHZ-88) though downregulating Wnt/β-catenin signaling pathway [128]. Consistently, Visweswaran et al. [129] also determined the downregulation of active β-catenin and Cyclin D1 (the major target proteins in the Wnt signaling pathway), and further reduced the expression of anti-apoptotic protein Bcl-xL.

### MSCs inhibit the tumor growth by impairing angiogenesis

The initiation, progression and development of tumor is closely associated with angiogenesis. Although it has been documented that MSCs possess superior pro-angiogenic potential, emerging evidence have shown that MSCs could also inhibit the tumor vascularization [130]. In a study, 106 MSCs were directly injected into subcutaneous melanoma of mice that had been growing for one week, and the results found the MSCs-induced apoptosis of tumor vascular system, resulting in significant inhibition of the tumor growth [131]. Moreover, Ho et al. [132] showed that the expression of platelet-derived growth factor (PDGF) and IL-1β in the conditioned medium of MSC/glioma co-culture were significantly downregulated, which seriously impaired the recruitment of endothelial progenitor cells (EPCs) and their ability to form endothelial tubes, which suggested that MSCs could inhibit the tumor angiogenesis by releasing anti-angiogenic factors.

### MSCs inhibit the tumor growth by regulating microRNA

In view of the abundant microRNA in MSC-secreted exosomes, several studies have evaluated the interaction between MSCs and tumor cells from the perspective of microRNA. Xu et al. [133] revealed that miR-133b in the exosomes of MSCs could inhibit the proliferation, invasion and migration of glioma cells by interfering Wnt/β-catenin signaling pathway though downregulation of EZH2. Besides, it has been demonstrated that the exosomes of BMSCs overexpressing miR-16-5p can inhibit the development of chromatic cancer (CRC) by downregulating ITGA2 [134]. Furthermore, it is reported that miR-205 in BMSCs exosomes inhibited the proliferation, invasion and migration of prostate cancer cells by targeting RHPN2, and promoted the apoptosis of cancer cells [135]. Similar study also found that overexpression of miR-15a in the exosomes derived from ADMSCs could impair the viability of CRC cells, and promote the apoptosis of CRC cells by downregulating KDM4B [136]; the subsequent study also suggested that UcMSCs-derived exosomal miR-15a-5p suppresses EMT and metastasis of cholangiocarcinoma through targeting downregulation of CHEK1 [137].

### MSCs inhibit the tumor growth by enhancing immunity

Concerned the present findings, the intricate interaction between MSCs and tumor cells makes researchers remain cautious about applying MSCs in the anticancer therapies. However, when we explore the relationship between MSCs and tumor, great attention should be paid to carefully and comprehensively analyzing the results obtained from the animal tumor model. Generally, the animal tumor models are established based on immunodeficient mice lacking normal immunologic function, then the MSCs-derived abundant nutritional cytokines seem to inevitably promote the growth of tumors [114, 125, 138]. However, the existence of normal immune system plays a key role in the outcomes of MSCs transplantation on tumor, and MSCs can inhibit tumor growth by activating immunocytes [139, 140]. Reportedly, under appropriate stimulation, MSC can secrete a large number of inflammatory factors, such as IL-1β, IFN-α, IFN-β, TNF-α and IFN-γ, which can effectively enhance the cytotoxicity of NK cells and the phagocytic function of macrophages [38, 138, 141]. Abumaree et al. have demonstrated that human decidua parietalis mesenchymal stem/multipotent stromal cells mediated activation and proliferation of resting NK cells was induced by IL-2 through activating NK cell receptors (NKG2D, CD69, NKp30 and NKp44) [142]. Moreover, in the model of colitis-associated colorectal cancer (CAC), intravenous injection of UcMSCs enhances the induction of Treg cells from naïve T cells and promotes the accumulation of Treg cells in the focal area, thus inhibiting the deterioration of colitis and delaying the progression of colon cancer [143].

### MSCs relieve chemotherapy-induced multiple organ damage

Notably, MSCs transplantation is still one of the most promising treatments for tissue regeneration and wound healing, and accumulating evidences suggest it as an ideal adjuvant for cancer treatment [144–146]. Studies have demonstrated that MSCs treatment can both improve chemotherapy-induced POF and relieve chemotherapy-induced multiple organ damage, such as cisplatin-induced kidney damage and hearing loss, doxorubicin-induced myocardial damage [147].

Cisplatin can be converted to toxic metabolites, which leads to acute renal injury and ototoxic hearing loss through increasing oxidative stress, reactive nitrogen substances, and further inducing apoptosis and inflammation [148]. Recently, Zhou et al. [147] have demonstrated that UcMSCs-derived exosomes can reverse cisplatin-induced acute renal injury by inhibiting p38MAPK signaling pathway through antioxidant stress. Besides, the interaction between 14 and 3-3ζ delivered by UcMSC-EVs and ATG16L could activate autophagy to prevent cisplatin-induced acute renal injury [149]. Furthermore, kang et al. have shown that both human amniotic epithelial cells (hAECs)and their derived EVs can protect the renal function from reducing cisplatin-induced mortality, serum creatinine and renal tubular injury score, which is contributed by inhibiting TNF-α/MAPK and caspase signaling pathways. Especially, they further revealed that hAECs or EVs neither promoted tumor growth nor impaired the therapeutic effect of cisplatin on tumor in xenografted mice of lung cancer [150]. Moreover, UcMSC-EVs could significantly improve the cis platin-induced hearing loss of mice and rescued the injured cochlear hair cells through upregulating the expression of GDN, mmu-miR-125a-5p, mmu-miR-125b-5p and mmu-miR127-5p in inner ear [151].

Additionally, Dox-induced cardiotoxicity including progressive cardiac remodeling cardiomyopathy and heart failure, seriously affect the life quality of patients and is life-threatening [152]. At present, various mechanisms of Dox-induced cardiomyocyte injury have been recognized, mainly including the disorder of redox homeostasis, the overproduction of ROS and the blocking of topoisomerase II-β [153]. Therefore, MSCs transplantation is taken as an optional treatment to protect against Dox-induced cardiotoxicity, and several studies have confirmed that MSCs is capable of protecting the heart from DOX-induced cytotoxicity in multiple ways. Lee et al. [154] observed that injection of MSCs-EVs from tail vein significantly improved the cardiac ejection fraction of DOX-treated mice, and the subsequent in vitro studies determined that MSCs-EVs could upregulate the expression of survivin through miR-199a-3p-Akt-Sp1/p53 signal pathway, contributing to protect cardiomyocytes against DOX-induced cytotoxicity. Importantly, further evidences indicated that MSCs-EVs play different roles in tumor cells and non-tumor cells, and MSCs-EVs treatment does not reduce the cytotoxicity of DOX on tumor cells [155]. In addition, hAFS/hMSC-CM also exhibited cardio-protective effects on mouse neonatal ventricular cardiomyocytes and their fibroblast counterpart, meanwhile, hAFS/hMSC-CM could improve DOX-induced the impairment of mitochondrial complex I activity, oxygen consumption and ATP synthesis [156].

Consequently, although only a few clinical studies have been performed to determine the therapeutic potential of MSCs and MSC-derived exosomes as anti-tumor treatment, the encouraging clinical outcomes have suggested the safety and efficacy of MSCs-based therapies for cancer patients. Simultaneously, MSCs treatment can effectively relieve cardiotoxicity, nephrotoxicity, ototoxicity and ovarian toxicity for the patients received chemotherapy, and finally improve their life quality and prolong their life span.

## Advantages and challenges of MenSC-based therapy

Although BMSCs, UcMSCs and adipose-derived stem cells (ADSCs) have been extensively studied and demonstrated to be viable options in numerous phase I/II clinical studies, several limitations including invasive procedures required for MSCs collection (BMSCs and ADSCs), low accessibility (UcMSCs) and a limited proliferative capacity (BMSCs) affect their clinical application [157–159]. Therefore, since the first report in 2007, MenSCs have gradually become a promising therapeutic option for various diseases without effective treatment due to their comprehensive advantages, such as a non-invasive protocol for their collection, their abundant source material, their stable donation, their superior proliferative capacity and their ability to be used for autologous transplantation [160]. A detailed introduction of MenSCs is beyond the scope of this review, and the reader can refer to existing publications for a comprehensive overview of their biological characteristics and therapeutic application.

### General characteristics

Generally, MenSCs can be successfully isolated from deciduous endometrium in menstrual blood which has often been treated as physiological waste collected in a non-invasive manner (menstrual cup), and subcultured MenSCs completely fulfill the international standard of MSCs, including a typical MSC morphology (spindle fibroblast-like morphology), classical MSC surface markers (positive for CD29, CD44, CD73, CD90 and CD105; negative for CD34, CD45 and human leukocyte antigen-DR isotype (HLA-DR), and multipotency (adipogenic, osteogenic, chondrogenic, neurogenic, and cardiogenic differentiation) [161, 162]. In addition to the above universal characteristics of other types of MSCs, MenSCs still have several unique characteristics, that increase their attractiveness and practicality: (1) The whole menstrual blood collection process is easy to perform at home, and MenSCs can be successfully isolated from samples stored at 4 °C for 72 h, which not only guarantees the privacy of donors but also facilitates express menstrual blood sample transportation [163]. (2) Menstrual blood samples can be collected monthly during the reproductive lives of healthy donors (20–30 years), and 40–80 ml of menstrual blood can be empirically collected during menstruation. Furthermore, based on our practice, approximately 0.5 × 10^5^ primary MenSCs can be isolated from 5 ml of menstrual blood, and these seeded MenSCs can proliferate and reach approximately 1 × 10^8^ cells after normal subculture to passage 3 [32]. Therefore, a healthy donor can provide a substantial quantity of MenSCs with a stable genetic background and biological activity, which not only satisfies the quantity requirement for clinical application but also guarantees the cell quality of MenSCs-based therapy. Because of the superior biological characteristics of MenSCs, six clinical studies of MenSCs-based therapies have been officially approved by the National Health Commission of the People's Republic of China.

### Unique advantages

Beside the advantages mentioned above, MenSCs transplantation is reasonably supposed to exhibit superior therapeutic effects for the female patients with reproductive dysfunction by virtue of the geographical relationship in which MenSCs originate from the endometrium [163, 164]. Therefore, the homologous and specific receptors expressed in MenSCs are likely to guide the stem cells reside in the reproductive organs (uterus and ovary) for a longer time, which provide superior opportunity for improving injured ovary from the perspective of time and space [165, 166]. Since 2014, Liu et al. [167] found that the MenSCs intraovarian injection into POF mice could survive at least 14 days in vivo and further differentiate into ovarian tissue-like cells in an ovarian microenvironment. Similarly, Lai et al. [168] also indicated that granulosa cells were capable of promoting MenSCs differentiate into oocyte-like cells in vitro. Moreover, the subsequent studies also confirmed that both MenSCs and MenSCs-derived exosomes have exhibited substantial improvement on ovarian function of POI/POF animal models, which likely result from improving the renewal of germline stem cells, promoting the proliferation of ovarian cells and follicle development, while inhibiting the follicle apoptosis and ovarian fibrosis [43, 81]. Promisingly, a phase I/II Clinical Trial launched at 2018 (TRN: IRCT20180619040147N2) confirmed the improvement of pregnancy rate and live birth rate in poor ovarian responders by intraovarian administration of autologous MenSCs, and resulted in 5 live births in MenSCs-treated group and one birth in control group [169].

As highlighted in the above-mentioned clinical trial, although autologous and allogeneic MSCs transplantation have their own merits and limitations, MenSCs-derived autologous transplantation may be the safer choice in terms of avoiding unwanted immune response and tissue rejection and reducing the potential foreign infections, especially for the younger female cancer patients who have fertility requirement [170, 171]. As similar with the oocyte or ovarian tissue cryopreservation for maintaining fertility of cancer patients before receiving chemotherapy and radiotherapy, the patients-derived MenSCs preparation and cryopreservation can be finished in advance through either collecting menstrual blood samples noninvasively or obtaining endometrial samples with a minimally invasive way (curettage) [163, 172]. Thereafter, the autologous MenSCs transplantation can be applied in cancer patients with chemotherapy as early as possible aiming to ameliorate the ovarian toxicity and systemic side effects caused by chemotherapeutic drugs. Currently, a retrospective study analyzed the outcomes of hospitalization for the patients presenting to the emergency department after hematopoietic stem cell transplantations (HSCT) which are taken as an effective treatment for hematological malignancies, and suggested that the patients received autologous HSCT exhibited lower risk of complications than that received allogeneic HSCT [173]. Simultaneously, a randomized, single-center, placebo-controlled phase I/II trial (NCT02513238) was performed to evaluate long-term safety of treatment with autologous MSCs in cancer patients with radiation-induced xerostomia, and the primary results not only confirmed the safety of autologous MSC therapy but also demonstrated that it significantly reduced xerostomia-related symptoms by improving unstimulated whole saliva flow rate. These clinical evidences provide solid support for the safety and efficacy of autologous MenSCs transplantation in diseases treatment [174].

### Challenges

To promote the clinical application of MenSCs and ensure a therapeutic effect after MenSCs transplantation, the whole process of MenSC-based therapy, including upstream (MenSC production), midstream (clinical parameters, such as the cell dosage, delivery route, and delivery time), and downstream (efficacy evaluation and mechanism analysis) factors, warrants greater consideration. First, standard MenSC production is the decisive factor for cell therapies; high-quality MenSCs are used in both preclinical and clinical studies and ensure therapeutic effects and reproducible results. Recently, “Expert consensus on the establishment of a standard MenSC bank” launched by our research group was published [175], which provides constructive and normative suggestions for MenSC isolation, culture, and cryopreservation. Second, both the cell dosage and delivery route play crucial roles in the therapeutic effect of MenSC transplantation, which might partly explain the unstable and even paradoxical results obtained in reported preclinical and clinical studies because of the different cell dosages and delivery routes used. Although the quantity of transplanted MSCs can be reasonably speculated to be positively correlated with the therapeutic effect on a disease, this positive correlation does not always exist, and several articles have reported opposite dose–response effects [176, 177]. Furthermore, in addition to conventional intravenous injection of MSCs, both ovarian injection under laparoscopy and arterial interventional injection are likely to effectively transplant MSCs into the injured ovary. However, the optimal quantity and delivery route for MenSCs transplantation still require further investigation in large animals before clinical application for chemotherapy-induced POI. Finally, MenSC-induced paracrine effects have been demonstrated to play a main role in improvements of injured tissue, and whether and how MenSC-induced paracrine effects restore the ovarian dysfunction caused by chemotherapy still require in-depth verification, which will provide support for the clinical application of MenSC-based therapy for chemotherapy-induced POI.

Moreover, in order to guarantee the therapeutic effects of MenSCs transplantation for the patients with POI, the source and quality of MenSCs is top priority and closely related with donors’ age and the physical condition. Firstly, the published data have indicated that the MenSCs isolated from middle-aged donors exhibit weaker potential for long-term subculture than that from young donors, and the genes involved in cell growth and development were significantly down-regulated with increase in donor age [178]. Importantly, it is reasonable to suppose that the characteristics and function of MenSCs isolated from patients are likely to be affected by their pathological state. Nikoo et al. [179] demonstrated that the stromal stem cells from patients with endometriosis (E-MenSCs) are distinct from that from non-endometriosis (NE-MenSCs) in terms of morphology, expression of surface markers, proliferation capacity. Consistently, Sahraei et al. [180] further determined that the differentially expressed genes between E-MenSCs and NE-MenSCs were mainly enriched in the signaling pathways of proliferation, migration and invasion (Cyclin D1, MMP-2 and MMP-9).

Additionally, besides the endometriosis, the autologous transplantation of MenSCs for patients with autoimmune diseases should also be carefully considered. Starc et al. [181] have determined the decreased immunomodulatory ability of MSCs isolated from primary immune-deficiencies patients. Similarly, another report also demonstrated that the MSCs isolated from lupus-like mice and systemic lupus erythematosus patients exhibited significant impairment in suppressing normal B cell proliferation and differentiation [182]. Although there is no published report directly demonstrate the abnormality of MenSCs isolated from patients with autoimmune diseases, the characteristics and function of MenSCs isolated from patients with autoimmune diseases is likely to exhibit abnormalities comparing with that from healthy donors. Because autoimmune diseases themselves are potential risk factor of resulting in POF, and it has been reported that 20% of POF patients suffering from concomitant autoimmune diseases (including adrenal disease, thyroid complications, and diabetes mellitus) [183], suggesting that it is not appropriate for patients with autoimmune diseases to perform autologous MSCs transplantation for immune intervention. Consequently, ensuring the MSCs quality plays an essential role for MenSCs derived therapeutic effects for POI patients, and it maybe optimal to cryopreserve the MenSCs at younger age with healthy physical state for donors, especially for the females with high risk of disease potential, but MenSCs cryopreservation related experimental studies and clinical trials are still needed.

## Conclusion

With the trend toward a younger onset of cancers, chemotherapy-induced POI has far-reaching effects on female patients. Especially for patients with fertility needs, the physiological and psychological changes that patients experience can cause many complications that place a serious burden on patients, their families, and society. However, when conventional treatments fail to effectively improve POI, MSC-based therapy has been preliminarily proven to be effective for treating ovarian injury. Due to their multifaceted advantages, MenSCs have exhibited promising therapeutic effects in various diseases and have yielded satisfactory results in basic research on ovarian function improvement. Therefore, under the premise of standardized production of MenSCs, optimizing clinical parameters such as the cell dose, cell delivery route, and timing of stem cell treatment will accelerate the clinical application of MenSCs and ensure their therapeutic effects.

## Data Availability

Data sharing is not applicable to this article as no new data were created or analyzed in this study.

## Abbreviations

POI: Premature ovarian insufficiency
MSC: Mesenchymal stem cell
MenSCs: Menstrual blood-derived endometrial stem cells
POF: Premature ovarian failure
FSH: Follicle-stimulating hormone
CTX: Cyclophosphamide
GCs: Granulosa cells
HRT: Hormone replacement therapy
iPSCs: Induced pluripotent stem cells
EPCs: Endothelial progenitor cells
PGCs: Primordial germ cells
ROS: Reactive oxygen species
RFP: Red fluorescent protein
AFCs: Amniotic fluid cells
UcMSC: Umbilical cord mesenchymal stem cell
UcMSC-Exos: UcMSC derived exosomes
BMSCs: Bone marrow stromal cells
PTEN: Phosphatase and tensin homolog deleted on chromosome 10
PI3K: Phosphatidylinositol-3-kinase
Akt: Protein kinase B
HO-1: Hemeoxygenase-1
DSBs: Double-strand breaks
AMSCs: Amniotic mesenchymal stem cells
VEGF: Vascular endothelial growth factor
MVs: Microvesicles
ADSCs: Adipose-derived mesenchymal stem cells
TGF-β: Tissue growth factor-β
HGF: Hepatocyte growth factor
IGF-1: Insulin-like growth factor-1
EGF: Epidermal growth factor
bFGF: Basic fibroblast growth factor
FOXO: Forkhead box O
Nrf-2: Nuclear factor E2–related factor 2
ARE: Antioxidant response element
BDNF: Brain-derived neurotrophic factor
LIF: Leukemia inhibitory factor
MT: Membrane receptor
JNK1: C-Jun N-terminal protein kinase 1
PCNA: Proliferating cell nuclear antigen
AMPK: 5′ AMP-activated protein kinase
ESCs: Embryonic stem cells
G-CSF: Granulocyte colony-stimulating factor
SCF: Stem cell factor
PDGF: Platelet-derived growth factor
PDCD4: Programmed cell death protein 4
HCCC: Human cholangiocarcinoma cells
CRC: Chromatic cancer
hAECs: Human amniotic epithelial cells
HSCT: Hematopoietic stem cell transplantations

## References

[1] Tsiligiannis S, Panay N, Stevenson JC (2019). Premature ovarian insufficiency and long-term health consequences. Curr Vasc Pharmacol.

[2] Goswami D, Conway GS (2005). Premature ovarian failure. Hum Reprod Update.

[3] van Dorp W, Haupt R, Anderson RA, Mulder RL, van den Heuvel-Eibrink MM, van Dulmen-den BE (2018). Reproductive function and outcomes in female survivors of childhood, adolescent, and young adult cancer: a review. J Clin Oncol.

[4] Wallace WH, Thomson AB, Kelsey TW (2003). The radiosensitivity of the human oocyte. Hum Reprod.

[5] Donnez J, Dolmans MM (2011). Preservation of fertility in females with haematological malignancy. Br J Haematol.

[6] Donnez J, Dolmans MM (2013). Fertility preservation in women. Nat Rev Endocrinol.

[7] Green DM, Kawashima T, Stovall M, Leisenring W, Sklar CA, Mertens AC (2009). Fertility of female survivors of childhood cancer: a report from the childhood cancer survivor study. J Clin Oncol.

[8] Roeca C, Dovey S, Polotsky AJ (2019). Recommendations for assessing ovarian health and fertility potential in survivors of childhood cancer. Maturitas.

[9] Bauer DC, Browner WS, Cauley JA, Orwoll ES, Scott JC, Black DM (1993). Factors associated with appendicular bone mass in older women. The study of osteoporotic fractures research group. Ann Intern Med.

[10] Ganz PA, Rowland JH, Desmond K, Meyerowitz BE, Wyatt GE (1998). Life after breast cancer: understanding women's health-related quality of life and sexual functioning. J Clin Oncol.

[11] Ganz PA, Greendale GA, Petersen L, Kahn B, Bower JE (2003). Breast cancer in younger women: reproductive and late health effects of treatment. J Clin Oncol.

[12] Cattoni A, Parissone F, Porcari I, Molinari S, Masera N, Franchi M (2021). Hormonal replacement therapy in adolescents and young women with chemo- or radio-induced premature ovarian insufficiency: practical recommendations. Blood Rev.

[13] Hickman LC, Llarena NC, Valentine LN, Liu X, Falcone T (2018). Preservation of gonadal function in women undergoing chemotherapy: a systematic review and meta-analysis of the potential role for gonadotropin-releasing hormone agonists. J Assist Reprod Genet.

[14] Beral V, Banks E, Reeves G (2002). Evidence from randomised trials on the long-term effects of hormone replacement therapy. Lancet.

[15] Li CI, Malone KE, Porter PL, Weiss NS, Tang MT, Cushing-Haugen KL (2003). Relationship between long durations and different regimens of hormone therapy and risk of breast cancer. JAMA.

[16] Harada M, Osuga Y (2019). Fertility preservation for female cancer patients. Int J Clin Oncol.

[17] Rodgers RJ (2019). Fertility preservation in breast cancer patients. Minerva Ginecol.

[18] Sheikhansari G, Aghebati-Maleki L, Nouri M, Jadidi-Niaragh F, Yousefi M (2018). Current approaches for the treatment of premature ovarian failure with stem cell therapy. Biomed Pharmacother.

[19] Chen L, Qu J, Cheng T, Chen X, Xiang C (2019). Menstrual blood-derived stem cells: toward therapeutic mechanisms, novel strategies, and future perspectives in the treatment of diseases. Stem Cell Res Ther.

[20] Bozorgmehr M, Gurung S, Darzi S, Nikoo S, Kazemnejad S, Zarnani AH (2020). Endometrial and menstrual blood mesenchymal stem/stromal cells: biological properties and clinical application. Front Cell Dev Biol.

[21] Takehara Y, Yabuuchi A, Ezoe K, Kuroda T, Yamadera R, Sano C (2013). The restorative effects of adipose-derived mesenchymal stem cells on damaged ovarian function. Lab Invest.

[22] Sun M, Wang S, Li Y, Yu L, Gu F, Wang C (2013). Adipose-derived stem cells improved mouse ovary function after chemotherapy-induced ovary failure. Stem Cell Res Ther.

[23] Fouad H, Sabry D, Elsetohy K, Fathy N (2016). Therapeutic efficacy of amniotic membrane stem cells and adipose tissue stem cells in rats with chemically induced ovarian failure. J Adv Res.

[24] Fu X, He Y, Wang X, Peng D, Chen X, Li X (2017). Overexpression of miR-21 in stem cells improves ovarian structure and function in rats with chemotherapy-induced ovarian damage by targeting PDCD4 and PTEN to inhibit granulosa cell apoptosis. Stem Cell Res Ther.

[25] Ling L, Feng X, Wei T, Wang Y, Wang Y, Zhang W (2017). Effects of low-intensity pulsed ultrasound (LIPUS)-pretreated human amnion-derived mesenchymal stem cell (hAD-MSC) transplantation on primary ovarian insufficiency in rats. Stem Cell Res Ther.

[26] Wang Z, Wang Y, Yang T, Li J, Yang X (2017). Study of the reparative effects of menstrual-derived stem cells on premature ovarian failure in mice. Stem Cell Res Ther.

[27] Chen X, Wang Q, Li X, Wang Q, Xie J, Fu X (2018). Heat shock pretreatment of mesenchymal stem cells for inhibiting the apoptosis of ovarian granulosa cells enhanced the repair effect on chemotherapy-induced premature ovarian failure. Stem Cell Res Ther.

[28] Li J, Yu Q, Huang H, Deng W, Cao X, Adu-Frimpong M (2018). Human chorionic plate-derived mesenchymal stem cells transplantation restores ovarian function in a chemotherapy-induced mouse model of premature ovarian failure. Stem Cell Res Ther.

[29] Feng P, Li P, Tan J (2019). Human menstrual blood-derived stromal cells promote recovery of premature ovarian insufficiency via regulating the ECM-dependent FAK/AKT signaling. Stem Cell Rev Rep.

[30] Sun B, Ma Y, Wang F, Hu L, Sun Y (2019). miR-644-5p carried by bone mesenchymal stem cell-derived exosomes targets regulation of p53 to inhibit ovarian granulosa cell apoptosis. Stem Cell Res Ther.

[31] Yang Z, Du X, Wang C, Zhang J, Liu C, Li Y (2019). Therapeutic effects of human umbilical cord mesenchymal stem cell-derived microvesicles on premature ovarian insufficiency in mice. Stem Cell Res Ther.

[32] Liu R, Zhang X, Fan Z, Wang Y, Yao G, Wan X (2019). Human amniotic mesenchymal stem cells improve the follicular microenvironment to recover ovarian function in premature ovarian failure mice. Stem Cell Res Ther.

[33] Huang B, Qian C, Ding C, Meng Q, Zou Q, Li H (2019). Fetal liver mesenchymal stem cells restore ovarian function in premature ovarian insufficiency by targeting MT1. Stem Cell Res Ther.

[34] Yang M, Lin L, Sha C, Li T, Zhao D, Wei H (2020). Bone marrow mesenchymal stem cell-derived exosomal miR-144-5p improves rat ovarian function after chemotherapy-induced ovarian failure by targeting PTEN. Lab Invest.

[35] Thabet E, Yusuf A, Abdelmonsif DA, Nabil I, Mourad G, Mehanna RA (2020). Extracellular vesicles miRNA-21: a potential therapeutic tool in premature ovarian dysfunction. Mol Hum Reprod.

[36] Cho J, Kim TH, Seok J, Jun JH, Park H, Kweon M (2021). Vascular remodeling by placenta-derived mesenchymal stem cells restores ovarian function in ovariectomized rat model via the VEGF pathway. Lab Invest.

[37] Seok J, Park H, Choi JH, Lim JY, Kim KG, Kim GJ (2020). Placenta-Derived Mesenchymal Stem Cells Restore the Ovary Function in an Ovariectomized Rat Model via an Antioxidant Effect. Antioxidants.

[38] Yin P, Gui L, Wang C, Yan J, Liu M, Ji L (2020). Targeted delivery of CXCL9 and OX40L by mesenchymal stem cells elicits potent antitumor immunity. Mol Ther.

[39] Yoon SY, Yoon JA, Park M, Shin EY, Jung S, Lee JE (2020). Recovery of ovarian function by human embryonic stem cell-derived mesenchymal stem cells in cisplatin-induced premature ovarian failure in mice. Stem Cell Res Ther.

[40] Ding C, Zhu L, Shen H, Lu J, Zou Q, Huang C (2020). Exosomal miRNA-17-5p derived from human umbilical cord mesenchymal stem cells improves ovarian function in premature ovarian insufficiency by regulating SIRT7. Stem Cells.

[41] Bahrehbar K, Rezazadeh Valojerdi M, Esfandiari F, Fathi R, Hassani SN, Baharvand H (2020). Human embryonic stem cell-derived mesenchymal stem cells improved premature ovarian failure. World J Stem Cells.

[42] Yamchi NN, Rahbarghazi R, Bedate AM, Mahdipour M, Nouri M, Khanbabaee R (2021). Menstrual blood CD146(+) mesenchymal stem cells reduced fibrosis rate in the rat model of premature ovarian failure. Cell Biochem Funct.

[43] Zhang S, Huang B, Su P, Chang Q, Li P, Song A (2021). Concentrated exosomes from menstrual blood-derived stromal cells improves ovarian activity in a rat model of premature ovarian insufficiency. Stem Cell Res Ther.

[44] Zhou Y, Zhou J, Xu X, Du F, Nie M, Hu L (2021). Matrigel/umbilical cord-derived mesenchymal stem cells promote granulosa cell proliferation and ovarian vascularization in a mouse model of premature ovarian failure. Stem Cells Dev.

[45] Lv X, Guan C, Li Y, Su X, Zhang L, Wang X (2021). Effects of single and multiple transplantations of human umbilical cord mesenchymal stem cells on the recovery of ovarian function in the treatment of premature ovarian failure in mice. J Ovarian Res.

[46] Deng T, He J, Yao Q, Wu L, Xue L, Wu M (2021). Human umbilical cord mesenchymal stem cells improve ovarian function in chemotherapy-induced premature ovarian failure mice through inhibiting apoptosis and inflammation via a paracrine mechanism. Reprod Sci.

[47] Wang J, Zhao Y, Zheng F, Ma N, Qin R, Qin W (2021). Activated human umbilical cord blood platelet-rich plasma enhances the beneficial effects of human umbilical cord mesenchymal stem cells in chemotherapy-induced POF rats. Stem Cells Int.

[48] Bahrehbar K, Gholami S, Nazari Z, Malakhond MK (2022). Embryonic stem cells-derived mesenchymal stem cells do not differentiate into ovarian cells but improve ovarian function in POF mice. Biochem Biophys Res Commun.

[49] El-Hayek S, Yang Q, Abbassi L, FitzHarris G, Clarke HJ (2018). Mammalian oocytes locally remodel follicular architecture to provide the foundation for germline-soma communication. Curr Biol..

[50] Havelock JC, Rainey WE, Carr BR (2004). Ovarian granulosa cell lines. Mol Cell Endocrinol.

[51] Wallace WH, Kelsey TW (2010). Human ovarian reserve from conception to the menopause. PLoS ONE.

[52] Albamonte MI, Albamonte MS, Stella I, Zuccardi L, Vitullo AD (2013). The infant and pubertal human ovary: balbiani's body-associated VASA expression, immunohistochemical detection of apoptosis-related BCL2 and BAX proteins, and DNA fragmentation. Hum Reprod.

[53] Chun EK, Jee BC, Kim JY, Kim SH, Moon SY (2014). Effect of imatinib coadministration on in vitro oocyte acquisition and subsequent embryo development in cyclophosphamide-treated mice. Reprod Sci.

[54] Bines J, Oleske DM, Cobleigh MA (1996). Ovarian function in premenopausal women treated with adjuvant chemotherapy for breast cancer. J Clin Oncol.

[55] Ben-Aharon I, Bar-Joseph H, Tzarfaty G, Kuchinsky L, Rizel S, Stemmer SM (2010). Doxorubicin-induced ovarian toxicity. Reprod Biol Endocrinol.

[56] Yuksel A, Bildik G, Senbabaoglu F, Akin N, Arvas M, Unal F (2015). The magnitude of gonadotoxicity of chemotherapy drugs on ovarian follicles and granulosa cells varies depending upon the category of the drugs and the type of granulosa cells. Hum Reprod.

[57] Overbeek A, van den Berg MH, van Leeuwen FE, Kaspers GJ, Lambalk CB, van Dulmen-den BE (2017). Chemotherapy-related late adverse effects on ovarian function in female survivors of childhood and young adult cancer: a systematic review. Cancer Treat Rev.

[58] Zhang T, He WH, Feng LL, Huang HG (2017). Effect of doxorubicin-induced ovarian toxicity on mouse ovarian granulosa cells. Regul Toxicol Pharmacol.

[59] Molina JR, Barton DL, Loprinzi CL (2005). Chemotherapy-induced ovarian failure: manifestations and management. Drug Saf.

[60] Morgan S, Anderson RA, Gourley C, Wallace WH, Spears N (2012). How do chemotherapeutic agents damage the ovary?. Hum Reprod Update.

[61] Ben-Aharon I, Shalgi R (2012). What lies behind chemotherapy-induced ovarian toxicity?. Reproduction.

[62] Codacci-Pisanelli G, Del Pup L, Del Grande M, Peccatori FA (2017). Mechanisms of chemotherapy-induced ovarian damage in breast cancer patients. Crit Rev Oncol Hematol.

[63] Spears N, Lopes F, Stefansdottir A, Rossi V, De Felici M, Anderson RA (2019). Ovarian damage from chemotherapy and current approaches to its protection. Hum Reprod Update.

[64] Eppig JJ (2018). Reproduction: oocytes call, granulosa cells connect. Curr Biol.

[65] Liu T, Huang Y, Guo L, Cheng W, Zou G (2012). CD44+/CD105+ human amniotic fluid mesenchymal stem cells survive and proliferate in the ovary long-term in a mouse model of chemotherapy-induced premature ovarian failure. Int J Med Sci.

[66] Zhang J, Yin H, Jiang H, Du X, Yang Z (2020). The protective effects of human umbilical cord mesenchymal stem cell-derived extracellular vesicles on cisplatin-damaged granulosa cells. Taiwan J Obstet Gynecol.

[67] Yin N, Wu C, Qiu J, Zhang Y, Bo L, Xu Y (2020). Protective properties of heme oxygenase-1 expressed in umbilical cord mesenchymal stem cells help restore the ovarian function of premature ovarian failure mice through activating the JNK/Bcl-2 signal pathway-regulated autophagy and upregulating the circulating of CD8(+)CD28(-) T cells. Stem Cell Res Ther.

[68] Soleimani R, Heytens E, Darzynkiewicz Z, Oktay K (2011). Mechanisms of chemotherapy-induced human ovarian aging: double strand DNA breaks and microvascular compromise. Aging.

[69] Liu M, Hales BF, Robaire B (2014). Effects of four chemotherapeutic agents, bleomycin, etoposide, cisplatin, and cyclophosphamide, on DNA damage and telomeres in a mouse spermatogonial cell line. Biol Reprod.

[70] Suh EK, Yang A, Kettenbach A, Bamberger C, Michaelis AH, Zhu Z (2006). p63 protects the female germ line during meiotic arrest. Nature.

[71] Gonfloni S, Di Tella L, Caldarola S, Cannata SM, Klinger FG, Di Bartolomeo C (2009). Inhibition of the c-Abl-TAp63 pathway protects mouse oocytes from chemotherapy-induced death. Nat Med.

[72] Xiao GY, Liu IH, Cheng CC, Chang CC, Lee YH, Cheng WT (2014). Amniotic fluid stem cells prevent follicle atresia and rescue fertility of mice with premature ovarian failure induced by chemotherapy. PLoS ONE.

[73] Gabr H, Rateb MA, El Sissy MH, Ahmed Seddiek H, Ali Abdelhameed Gouda S (2016). The effect of bone marrow-derived mesenchymal stem cells on chemotherapy induced ovarian failure in albino rats. Microsc Res Tech.

[74] Potente M, Gerhardt H, Carmeliet P (2011). Basic and therapeutic aspects of angiogenesis. Cell.

[75] Delgado-Rosas F, Gaytan M, Morales C, Gomez R, Gaytan F (2009). Superficial ovarian cortex vascularization is inversely related to the follicle reserve in normal cycling ovaries and is increased in polycystic ovary syndrome. Hum Reprod.

[76] Bar-Joseph H, Ben-Aharon I, Tzabari M, Tsarfaty G, Stemmer SM, Shalgi R (2011). In vivo bioimaging as a novel strategy to detect doxorubicin-induced damage to gonadal blood vessels. PLoS ONE.

[77] Dubreuil G (1946). The cortical stroma of the female ovary; its various modes of functional adaptation. Bull Histol Appl Physiol Pathol Tech Microsc.

[78] Craig JM (1967). Ovarian cortical stroma, a steroid-dependent tissue. Am J Obstet Gynecol.

[79] Meirow D, Dor J, Kaufman B, Shrim A, Rabinovici J, Schiff E (2007). Cortical fibrosis and blood-vessels damage in human ovaries exposed to chemotherapy. Potential mechanisms of ovarian injury. Hum Reprod.

[80] Marcello MF, Nuciforo G, Romeo R, Di Dino G, Russo I, Russo A (1990). Structural and ultrastructural study of the ovary in childhood leukemia after successful treatment. Cancer.

[81] Lai D, Wang F, Yao X, Zhang Q, Wu X, Xiang C (2015). Human endometrial mesenchymal stem cells restore ovarian function through improving the renewal of germline stem cells in a mouse model of premature ovarian failure. J Transl Med.

[82] Shojafar E, Soleimani Mehranjani M, Shariatzadeh SMA (2018). Adipose-derived mesenchymal stromal cell transplantation at the graft site improves the structure and function of autografted mice ovaries: a stereological and biochemical analysis. Cytotherapy.

[83] Sies H (2015). Oxidative stress: a concept in redox biology and medicine. Redox Biol.

[84] Ravid A, Rocker D, Machlenkin A, Rotem C, Hochman A, Kessler-Icekson G (1999). 1,25-Dihydroxyvitamin D3 enhances the susceptibility of breast cancer cells to doxorubicin-induced oxidative damage. Cancer Res.

[85] Roberts I (2004). Mesenchymal stem cells. Vox Sang.

[86] Oh HM, Yu CR, Dambuza I, Marrero B, Egwuagu CE (2012). STAT3 protein interacts with class O Forkhead transcription factors in the cytoplasm and regulates nuclear/cytoplasmic localization of FoxO1 and FoxO3a proteins in CD4(+) T cells. J Biol Chem.

[87] Lapp DW, Zhang SS, Barnstable CJ (2014). Stat3 mediates LIF-induced protection of astrocytes against toxic ROS by upregulating the UPC2 mRNA pool. Glia.

[88] Foxton R, Osborne A, Martin KR, Ng YS, Shima DT (2016). Distal retinal ganglion cell axon transport loss and activation of p38 MAPK stress pathway following VEGF-A antagonism. Cell Death Dis.

[89] Ryu BJ, Han JW, Kim RH, Yun S, Kim TH, Hur SE, et al. Activation of NOD-1/JNK/IL-8 signal axis in decidual stromal cells facilitates trophoblast invasion. Am J Reprod Immunol. 2017;78(2). 10.1111/aji.12672 28328096

[90] Ding C, Zou Q, Wang F, Wu H, Wang W, Li H (2018). HGF and BFGF secretion by human adipose-derived stem cells improves ovarian function during natural aging via activation of the SIRT1/FOXO1 signaling pathway. Cell Physiol Biochem.

[91] Yu C, Zhang X, Wang L, Liu Y, Li N, Li M (2018). Interleukin-6 regulates expression of Fos and Jun genes to affect the development of mouse preimplantation embryos. J Obstet Gynaecol Res.

[92] Zhao X, Du F, Liu X, Ruan Q, Wu Z, Lei C (2019). Brain-derived neurotrophic factor (BDNF) is expressed in buffalo (Bubalus bubalis) ovarian follicles and promotes oocyte maturation and early embryonic development. Theriogenology.

[93] Jang H, Lee OH, Lee Y, Yoon H, Chang EM, Park M (2016). Melatonin prevents cisplatin-induced primordial follicle loss via suppression of PTEN/AKT/FOXO3a pathway activation in the mouse ovary. J Pineal Res.

[94] Richards M, Fong CY, Bongso A (2010). Comparative evaluation of different in vitro systems that stimulate germ cell differentiation in human embryonic stem cells. Fertil Steril.

[95] Kang Y, Cheng MJ, Xu CJ (2011). Secretion of oestrogen from murine-induced pluripotent stem cells co-cultured with ovarian granulosa cells in vitro. Cell Biol Int.

[96] Liu T, Qin W, Huang Y, Zhao Y, Wang J (2013). Induction of estrogen-sensitive epithelial cells derived from human-induced pluripotent stem cells to repair ovarian function in a chemotherapy-induced mouse model of premature ovarian failure. DNA Cell Biol.

[97] Manshadi MD, Navid S, Hoshino Y, Daneshi E, Noory P, Abbasi M (2019). The effects of human menstrual blood stem cells-derived granulosa cells on ovarian follicle formation in a rat model of premature ovarian failure. Microsc Res Tech.

[98] Zhang Q, Bu S, Sun J, Xu M, Yao X, He K (2017). Paracrine effects of human amniotic epithelial cells protect against chemotherapy-induced ovarian damage. Stem Cell Res Ther.

[99] Haynesworth SE, Baber MA, Caplan AI (1996). Cytokine expression by human marrow-derived mesenchymal progenitor cells in vitro: effects of dexamethasone and IL-1 alpha. J Cell Physiol.

[100] Liu CH, Hwang SM (2005). Cytokine interactions in mesenchymal stem cells from cord blood. Cytokine.

[101] Choi MR, Kim HY, Park JY, Lee TY, Baik CS, Chai YG (2010). Selection of optimal passage of bone marrow-derived mesenchymal stem cells for stem cell therapy in patients with amyotrophic lateral sclerosis. Neurosci Lett.

[102] Volarevic V, Gazdic M, Simovic Markovic B, Jovicic N, Djonov V, Arsenijevic N (2017). Mesenchymal stem cell-derived factors: Immuno-modulatory effects and therapeutic potential. BioFactors.

[103] Shin SY, Lee JY, Lee E, Choi J, Yoon BK, Bae D (2006). Protective effect of vascular endothelial growth factor (VEGF) in frozen-thawed granulosa cells is mediated by inhibition of apoptosis. Eur J Obstet Gynecol Reprod Biol.

[104] Mao J, Smith MF, Rucker EB, Wu GM, McCauley TC, Cantley TC (2004). Effect of epidermal growth factor and insulin-like growth factor I on porcine preantral follicular growth, antrum formation, and stimulation of granulosal cell proliferation and suppression of apoptosis in vitro. J Anim Sci.

[105] Uzumcu M, Pan Z, Chu Y, Kuhn PE, Zachow R (2006). Immunolocalization of the hepatocyte growth factor (HGF) system in the rat ovary and the anti-apoptotic effect of HGF in rat ovarian granulosa cells in vitro. Reproduction.

[106] Mori S, Hatori N, Kawaguchi N, Hamada Y, Shih TC, Wu CY (2017). The integrin-binding defective FGF2 mutants potently suppress FGF2 signalling and angiogenesis. Biosci Rep.

[107] van Niel G, D'Angelo G, Raposo G (2018). Shedding light on the cell biology of extracellular vesicles. Nat Rev Mol Cell Biol.

[108] Xiao GY, Cheng CC, Chiang YS, Cheng WT, Liu IH, Wu SC (2016). Exosomal miR-10a derived from amniotic fluid stem cells preserves ovarian follicles after chemotherapy. Sci Rep.

[109] Murphy MB, Moncivais K, Caplan AI (2013). Mesenchymal stem cells: environmentally responsive therapeutics for regenerative medicine. Exp Mol Med.

[110] Lo Sicco C, Reverberi D, Balbi C, Ulivi V, Principi E, Pascucci L (2017). Mesenchymal stem cell-derived extracellular vesicles as mediators of anti-inflammatory effects: endorsement of macrophage polarization. Stem Cells Transl Med.

[111] Zhao X, Zhao Y, Sun X, Xing Y, Wang X, Yang Q (2020). Immunomodulation of MSCs and MSC-derived extracellular vesicles in osteoarthritis. Front Bioeng Biotechnol.

[112] Qiao L, Xu Z, Zhao T, Zhao Z, Shi M, Zhao RC (2008). Suppression of tumorigenesis by human mesenchymal stem cells in a hepatoma model. Cell Res.

[113] Wakitani S, Okabe T, Horibe S, Mitsuoka T, Saito M, Koyama T (2011). Safety of autologous bone marrow-derived mesenchymal stem cell transplantation for cartilage repair in 41 patients with 45 joints followed for up to 11 years and 5 months. J Tissue Eng Regen Med.

[114] Harrell CR, Volarevic A, Djonov VG, Jovicic N, Volarevic V (2021). Mesenchymal stem cell: a friend or foe in anti-tumor immunity. Int J Mol Sci.

[115] Zhuang WZ, Lin YH, Su LJ, Wu MS, Jeng HY, Chang HC (2021). Mesenchymal stem/stromal cell-based therapy: mechanism, systemic safety and biodistribution for precision clinical applications. J Biomed Sci.

[116] Liang W, Chen X, Zhang S, Fang J, Chen M, Xu Y (2021). Mesenchymal stem cells as a double-edged sword in tumor growth: focusing on MSC-derived cytokines. Cell Mol Biol Lett.

[117] Zacharek A, Chen J, Cui X, Li A, Li Y, Roberts C (2007). Angiopoietin1/Tie2 and VEGF/Flk1 induced by MSC treatment amplifies angiogenesis and vascular stabilization after stroke. J Cereb Blood Flow Metab.

[118] Du WJ, Chi Y, Yang ZX, Li ZJ, Cui JJ, Song BQ (2016). Heterogeneity of proangiogenic features in mesenchymal stem cells derived from bone marrow, adipose tissue, umbilical cord, and placenta. Stem Cell Res Ther.

[119] Beckermann BM, Kallifatidis G, Groth A, Frommhold D, Apel A, Mattern J (2008). VEGF expression by mesenchymal stem cells contributes to angiogenesis in pancreatic carcinoma. Br J Cancer.

[120] Huang WH, Chang MC, Tsai KS, Hung MC, Chen HL, Hung SC (2013). Mesenchymal stem cells promote growth and angiogenesis of tumors in mice. Oncogene.

[121] Sanchez C, Oskowitz A, Pochampally RR (2009). Epigenetic reprogramming of IGF1 and leptin genes by serum deprivation in multipotential mesenchymal stromal cells. Stem Cells.

[122] Ma OK, Chan KH (2016). Immunomodulation by mesenchymal stem cells: Interplay between mesenchymal stem cells and regulatory lymphocytes. World J Stem Cells.

[123] Poggi A, Varesano S, Zocchi MR (2018). How to hit mesenchymal stromal cells and make the tumor microenvironment immunostimulant rather than immunosuppressive. Front Immunol.

[124] Ma M, Ye JY, Deng R, Dee CM, Chan GC (2011). Mesenchymal stromal cells may enhance metastasis of neuroblastoma via SDF-1/CXCR4 and SDF-1/CXCR7 signaling. Cancer Lett.

[125] Yu JL, Chan S, Fung MK, Chan GC (2021). Mesenchymal stem cells accelerated growth and metastasis of neuroblastoma and preferentially homed towards both primary and metastatic loci in orthotopic neuroblastoma model. BMC Cancer.

[126] Lan T, Luo M, Wei X (2021). Mesenchymal stem/stromal cells in cancer therapy. J Hematol Oncol.

[127] Liu J, Han G, Liu H, Qin C (2013). Suppression of cholangiocarcinoma cell growth by human umbilical cord mesenchymal stem cells: a possible role of Wnt and Akt signaling. PLoS ONE.

[128] Visweswaran M, Arfuso F, Dilley RJ, Newsholme P, Dharmarajan A (2018). The inhibitory influence of adipose tissue-derived mesenchymal stem cell environment and Wnt antagonism on breast tumour cell lines. Int J Biochem Cell Biol.

[129] Otsu K, Das S, Houser SD, Quadri SK, Bhattacharya S, Bhattacharya J (2009). Concentration-dependent inhibition of angiogenesis by mesenchymal stem cells. Blood.

[130] Kucerova L, Matuskova M, Hlubinova K, Altanerova V, Altaner C (2010). Tumor cell behaviour modulation by mesenchymal stromal cells. Mol Cancer.

[131] Ho IA, Toh HC, Ng WH, Teo YL, Guo CM, Hui KM (2013). Human bone marrow-derived mesenchymal stem cells suppress human glioma growth through inhibition of angiogenesis. Stem Cells.

[132] Xu H, Zhao G, Zhang Y, Jiang H, Wang W, Zhao D (2019). Mesenchymal stem cell-derived exosomal microRNA-133b suppresses glioma progression via Wnt/beta-catenin signaling pathway by targeting EZH2. Stem Cell Res Ther.

[133] Xu Y, Shen L, Li F, Yang J, Wan X, Ouyang M (2019). microRNA-16-5p-containing exosomes derived from bone marrow-derived mesenchymal stem cells inhibit proliferation, migration, and invasion, while promoting apoptosis of colorectal cancer cells by downregulating ITGA2. J Cell Physiol.

[134] Jiang S, Mo C, Guo S, Zhuang J, Huang B, Mao X (2019). Human bone marrow mesenchymal stem cells-derived microRNA-205-containing exosomes impede the progression of prostate cancer through suppression of RHPN2. J Exp Clin Cancer Res.

[135] Liu L, Yu T, Jin Y, Mai W, Zhou J, Zhao C (2021). MicroRNA-15a carried by mesenchymal stem cell-derived extracellular vesicles inhibits the immune evasion of colorectal cancer cells by regulating the KDM4B/HOXC4/PD-L1 Axis. Front Cell Dev Biol.

[136] Li N, Wang B (2022). Suppressive effects of umbilical cord mesenchymal stem cell-derived exosomal miR-15a-5p on the progression of cholangiocarcinoma by inhibiting CHEK1 expression. Cell Death Discov.

[137] Chen J, Ji T, Wu D, Jiang S, Zhao J, Lin H (2019). Human mesenchymal stem cells promote tumor growth via MAPK pathway and metastasis by epithelial mesenchymal transition and integrin alpha5 in hepatocellular carcinoma. Cell Death Dis.

[138] Abumaree MH, Bahattab E, Alsadoun A, Al Dosaimani A, Abomaray FM, Khatlani T (2018). Characterization of the interaction between human decidua parietalis mesenchymal stem/stromal cells and natural killer cells. Stem Cell Res Ther.

[139] Rivera-Cruz CM, Shearer JJ, Figueiredo Neto M, Figueiredo ML (2017). The immunomodulatory effects of mesenchymal stem cell polarization within the tumor microenvironment niche. Stem Cells Int.

[140] Francois S, Usunier B, Forgue-Lafitte ME, L'Homme B, Benderitter M, Douay L (2019). Mesenchymal stem cell administration attenuates colon cancer progression by modulating the immune component within the colorectal tumor microenvironment. Stem Cells Transl Med.

[141] Jing W, Chen Y, Lu L, Hu X, Shao C, Zhang Y (2014). Human umbilical cord blood-derived mesenchymal stem cells producing IL15 eradicate established pancreatic tumor in syngeneic mice. Mol Cancer Ther.

[142] Tang RJ, Shen SN, Zhao XY, Nie YZ, Xu YJ, Ren J (2015). Mesenchymal stem cells-regulated Treg cells suppress colitis-associated colorectal cancer. Stem Cell Res Ther.

[143] Liu W, Yu M, Xie D, Wang L, Ye C, Zhu Q (2020). Melatonin-stimulated MSC-derived exosomes improve diabetic wound healing through regulating macrophage M1 and M2 polarization by targeting the PTEN/AKT pathway. Stem Cell Res Ther.

[144] Hendijani F (2015). Human mesenchymal stromal cell therapy for prevention and recovery of chemo/radiotherapy adverse reactions. Cytotherapy.

[145] Yang J, Chen Z, Pan D, Li H, Shen J (2020). Umbilical cord-derived mesenchymal stem cell-derived exosomes combined pluronic F127 hydrogel promote chronic diabetic wound healing and complete skin regeneration. Int J Nanomedicine.

[146] Levoux J, Prola A, Lafuste P, Gervais M, Chevallier N, Koumaiha Z (2021). Platelets facilitate the wound-healing capability of mesenchymal stem cells by mitochondrial transfer and metabolic reprogramming. Cell Metab.

[147] Zhou Y, Xu H, Xu W, Wang B, Wu H, Tao Y (2013). Exosomes released by human umbilical cord mesenchymal stem cells protect against cisplatin-induced renal oxidative stress and apoptosis in vivo and in vitro. Stem Cell Res Ther.

[148] Ma Z, Hu X, Ding HF, Zhang M, Huo Y, Dong Z (2022). Single-nucleus transcriptional profiling of chronic kidney disease after cisplatin nephrotoxicity. Am J Pathol.

[149] Jia H, Liu W, Zhang B, Wang J, Wu P, Tandra N (2018). HucMSC exosomes-delivered 14-3-3zeta enhanced autophagy via modulation of ATG16L in preventing cisplatin-induced acute kidney injury. Am J Transl Res.

[150] Kang X, Chen Y, Xin X, Liu M, Ma Y, Ren Y (2021). Human amniotic epithelial cells and their derived exosomes protect against cisplatin-induced acute kidney injury without compromising its antitumor activity in mice. Front Cell Dev Biol.

[151] Tsai SC, Yang KD, Chang KH, Lin FC, Chou RH, Li MC (2021). Umbilical cord mesenchymal stromal cell-derived exosomes rescue the loss of outer hair cells and repair cochlear damage in cisplatin-injected mice. Int J Mol Sci.

[152] Anker MS, Hadzibegovic S, Lena A, Belenkov Y, Bergler-Klein J, de Boer RA (2019). Recent advances in cardio-oncology: a report from the 'heart failure association 2019 and world congress on acute heart failure 2019'. ESC Heart Fail.

[153] Cui N, Wu F, Lu WJ, Bai R, Ke B, Liu T (2019). Doxorubicin-induced cardiotoxicity is maturation dependent due to the shift from topoisomerase IIalpha to IIbeta in human stem cell derived cardiomyocytes. J Cell Mol Med.

[154] Lee JY, Chung J, Byun Y, Kim KH, An SH, Kwon K (2021). Mesenchymal stem cell-derived small extracellular vesicles protect cardiomyocytes from doxorubicin-induced cardiomyopathy by upregulating survivin expression via the miR-199a-3p-Akt-Sp1/p53 signaling pathway. Int J Mol Sci.

[155] Serras AS, Camoes SP, Antunes B, Costa VM, Dionisio F, Yazar V (2021). The secretome of human neonatal mesenchymal stem cells modulates doxorubicin-induced cytotoxicity: impact in non-tumor cells. Int J Mol Sci.

[156] Villa F, Bruno S, Costa A, Li M, Russo M, Cimino J (2021). The human fetal and adult stem cell secretome can exert cardioprotective paracrine effects against cardiotoxicity and oxidative stress from cancer treatment. Cancers.

[157] Riester SM, Denbeigh JM, Lin Y, Jones DL, de Mooij T, Lewallen EA (2017). Safety studies for use of adipose tissue-derived mesenchymal stromal/stem cells in a rabbit model for osteoarthritis to support a phase I clinical trial. Stem Cells Transl Med.

[158] Reinders MEJ, Groeneweg KE, Hendriks SH, Bank JR, Dreyer GJ, de Vries APJ (2021). Autologous bone marrow-derived mesenchymal stromal cell therapy with early tacrolimus withdrawal: The randomized prospective, single-center, open-label TRITON study. Am J Transplant.

[159] Meng F, Xu R, Wang S, Xu Z, Zhang C, Li Y (2020). Human umbilical cord-derived mesenchymal stem cell therapy in patients with COVID-19: a phase 1 clinical trial. Signal Transduct Target Ther.

[160] Meng X, Ichim TE, Zhong J, Rogers A, Yin Z, Jackson J (2007). Endometrial regenerative cells: a novel stem cell population. J Transl Med.

[161] Zemel'ko VI, Grinchuk TM, Domnina AP, Artsybasheva IV, Zenin VV, Kirsanov AA (2011). Multipotent mesenchymal stem cells of desquamated endometrium: isolation, characterization and use as feeder layer for maintenance of human embryonic stem cell lines. Tsitologiia.

[162] Lin J, Xiang D, Zhang JL, Allickson J, Xiang C (2011). Plasticity of human menstrual blood stem cells derived from the endometrium. J Zhejiang Univ Sci B.

[163] Liu Y, Niu R, Yang F, Yan Y, Liang S, Sun Y (2018). Biological characteristics of human menstrual blood-derived endometrial stem cells. J Cell Mol Med.

[164] Gargett CE, Schwab KE, Deane JA (2016). Endometrial stem/progenitor cells: the first 10 years. Hum Reprod Update.

[165] Tempest N, Maclean A, Hapangama DK (2018). Endometrial stem cell markers: current concepts and unresolved questions. Int J Mol Sci.

[166] Lv H, Hu Y, Cui Z, Jia H (2018). Human menstrual blood: a renewable and sustainable source of stem cells for regenerative medicine. Stem Cell Res Ther.

[167] Liu T, Huang Y, Zhang J, Qin W, Chi H, Chen J (2014). Transplantation of human menstrual blood stem cells to treat premature ovarian failure in mouse model. Stem Cells Dev.

[168] Lai D, Guo Y, Zhang Q, Chen Y, Xiang C (2016). Differentiation of human menstrual blood-derived endometrial mesenchymal stem cells into oocyte-like cells. Acta Biochim Biophys Sin.

[169] Zafardoust S, Kazemnejad S, Darzi M, Fathi-Kazerooni M, Rastegari H, Mohammadzadeh A (2020). Improvement of pregnancy rate and live birth rate in poor ovarian responders by intraovarian administration of autologous menstrual blood derived-mesenchymal stromal cells: phase I/II clinical trial. Stem Cell Rev Rep.

[170] Shah K, Shah N, Ghassemi F, Ly C, George T, Lutz C (2022). Alloreactivity of allogeneic mesenchymal stem/stromal cells and other cellular therapies: a concise review. Stem Cells Int.

[171] Durand N, Zubair AC (2022). Autologous versus allogeneic mesenchymal stem cell therapy: the pros and cons. Surgery.

[172] Liu Y, Liang S, Yang F, Sun Y, Niu L, Ren Y (2020). Biological characteristics of endometriotic mesenchymal stem cells isolated from ectopic lesions of patients with endometriosis. Stem Cell Res Ther.

[173] Spoerl S, Hendlmeier C, Hapfelmeier A, Wildgruber M, Schmid RM, Peschel C (2017). Characteristics and outcome of patients presenting to the emergency department after autologous/allogeneic stem cell transplantation. Eur J Emerg Med.

[174] Lynggaard CD, Gronhoj C, Jensen SB, Christensen R, Specht L, Andersen E (2022). Long-term safety of treatment with autologous mesenchymal stem cells in patients with radiation-induced xerostomia: primary results of the MESRIX phase I/II randomized trial. Clin Cancer Res.

[175] Stem Cell Committee of Henan Society of Cell Biology. Professional Committee of Clinical Data and Sample Database of Chinese Association of Research Hospitals. Expert consensus on the establishment of a standard menstrual blood-derived endometrial stem cell bank. Journal of Xinxiang Medical University. 2020;9: 801–2.

[176] Losordo DW, Henry TD, Davidson C, Sup Lee J, Costa MA, Bass T (2011). Intramyocardial, autologous CD34+ cell therapy for refractory angina. Circ Res.

[177] Hare JM, Fishman JE, Gerstenblith G, DiFede Velazquez DL, Zambrano JP, Suncion VY (2012). Comparison of allogeneic vs autologous bone marrow-derived mesenchymal stem cells delivered by transendocardial injection in patients with ischemic cardiomyopathy: the POSEIDON randomized trial. JAMA.

[178] Chen J, Du X, Chen Q, Xiang C (2015). Effects of donors' age and passage number on the biological characteristics of menstrual blood-derived stem cells. Int J Clin Exp Pathol.

[179] Nikoo S, Ebtekar M, Jeddi-Tehrani M, Shervin A, Bozorgmehr M, Vafaei S (2014). Menstrual blood-derived stromal stem cells from women with and without endometriosis reveal different phenotypic and functional characteristics. Mol Hum Reprod.

[180] Sahraei SS, Davoodi Asl F, Kalhor N, Sheykhhasan M, Fazaeli H, Moud SS (2022). A comparative study of gene expression in menstrual blood-derived stromal cells between endometriosis and healthy women. Biomed Res Int.

[181] Starc N, Ingo D, Conforti A, Rossella V, Tomao L, Pitisci A (2017). Biological and functional characterization of bone marrow-derived mesenchymal stromal cells from patients affected by primary immunodeficiency. Sci Rep.

[182] Che N, Li X, Zhang L, Liu R, Chen H, Gao X (2014). Impaired B cell inhibition by lupus bone marrow mesenchymal stem cells is caused by reduced CCL2 expression. J Immunol.

[183] Santoro N (2001). Research on the mechanisms of premature ovarian failure. J Soc Gynecol Investig.

